# Robust prediction of glioma prognosis by hypoxia-induced ferroptosis genes: VEGFA-XBP1 co-expression for salvage therapy

**DOI:** 10.1080/15384047.2025.2529643

**Published:** 2025-07-07

**Authors:** Zhou Liwei, Lu Hanwen, Zhao Wenpeng, Yu Weijie, Chen Sifang, Zhang Bingchang, Li Zhangyu, Gao Xin, Li Wenhua, Mao Jianyao, Xie Yuanyuan, Tan Guowei, Wang Zhanxiang

**Affiliations:** a Department of Neurosurgery, Xiamen Key Laboratory of Brain Center, The First Affiliated Hospital of Xiamen University, Xiamen, China; b The Department of Neuroscience, Institute of Neurosurgery, School of Medicine, Xiamen University, Xiamen, China; c The School Of Clinical Medicine, Fujian Medical University, Fuzhou, China

**Keywords:** Hypoxia, ferroptosis, glioma, immunity, prognosis

## Abstract

Hypoxia as a hallmark of solid malignancies compromises therapeutic efficacy and prognosis. This study deciphers the functional role of hypoxia-induced ferroptosis in glioma prognosis. Hypoxia-related transcripts and ferroptosis markers were curated from public databases. ConsensusClusterPlus identify hypoxia-based molecular subtypes, while LASSO-penalized Cox regression integrated with limma-based differential expression analysis screened prognostic ferroptosis genes. Subsequent risk modeling was validated against clinical parameters and extended through nomogram construction. Protein-protein interaction networks centered on HIF-1αidentified high-confidence interactors, with parallel immune correlation analysis completing the systems-level investigation.Based on 27 hypoxia-associated genes, we stratified samples into three distinct hypoxic clusters. Differential analysis of 123 ferroptosis markers across clusters, combined with univariate Cox regression and LASSO regression, identified 23 hypoxia-induced ferroptosis genes for constructing a prognostic model. The model demonstrated robust predictive accuracy with AUC values of 0.80 (1-year), 0.86 (3-year), and 0.86 (5-year). GSEA revealed significant enrichment in ECM-receptor interactions, focal adhesion, JAK-STAT signaling, and p53 signaling pathways, suggesting their involvement in hypoxia-induced ferroptosis. Our risk model significantly outperformed conventional clinical parameters (pathology, grade, age, primary/recurrent status). Protein-protein interaction analysis incorporating HIF-1αand the 23-model genes identified XBP1 and VEGFA co-expression as significant positive prognostic factor. The immune infiltration analysis further indicated that M0 macrophages may participate in the regulation of the prognosis of VEGFA-XBP1.Hypoxia-induced ferroptosis modulation emerges as a prognostic factor in gliomas, with XBP1 and VEGFA representing druggable nodes for novel combination therapies.

## Introduction

Hypoxia is a characteristic feature of various solid tumors, including gliomas. Within this context, hypoxia promotes cancer cell growth by triggering a multitude of downstream target genes. These genes are responsible for uncontrolled angiogenesis of small blood vessels or epithelial-mesenchymal transition, as well as numerous other events associated with tumor progression. These hypoxia-driven events are correlated with a poor prognosis.^1–4^ Gliomas represent the most prevalent primary CNS malignancy, exhibiting accelerated proliferation and intensified hypoxia with ascending histological grade. Glioblastoma (GBM) exhibits a median survival of only 15 months despite aggressive multimodal therapy including surgery, radiation, and chemotherapy.^5^ As the primary hypoxia-responsive transcription factor, HIF-1α drives tumor progression by promoting angiogenesis, facilitating extracellular matrix (ECM) degradation via matrix metalloproteinases (MMPs), and enhancing metastatic dissemination.^6^ Hypoxia further drives the induction of glioma stem cells (GSCs), which confers therapeutic resistance and elevates the risk of recurrence, thereby establishing hypoxia as a critical prognostic determinant in gliomas.^7^

Ferroptosis, an iron-dependent, lipid peroxidation-driven cell death, involves dysregulated iron metabolism, antioxidant system failure, and lipid peroxide accumulation in tumors. It exhibits a “double-edged sword” effect on survival: moderate induction clears tumor cells improving prognosis, while chronic ferroptosis promotes metastasis, immunosuppression, therapy resistance, and reduced survival.^8,9^ Therapeutic resistance is pharmacologically reversible via precision ferroptosis intervention.^10^ In gliomas, ferroptosis-resistant tumor cells can adapt to harsh microenvironments, including hypoxia and nutrient deprivation, while simultaneously exhibiting resistance to chemotherapy and radiotherapy.^11^ Moreover, elevated expression of ferroptosis suppressor genes can promote the transformation of glioma cells into GSCs.^12^ Although ferroptosis has been implicated in promoting glioma aggressiveness and therapeutic resistance, its underlying mechanisms remain incompletely understood. Consequently, targeting this process to effectively induce ferroptosis represents a promising novel therapeutic strategy to overcome therapeutic resistance and improve clinical outcomes in glioma patients.

Hypoxia, a hallmark of the tumor microenvironment, profoundly modulates key cell death pathways, such as apoptosis and autophagy.^13,14^ However, the impact of hypoxia on ferroptosis remains unclear. Notably, studies in HT-1080 fibrosarcoma cells suggest that HIF-1αinhibits ferroptosis by upregulating fatty acid-binding proteins 3 and 7 (FABP3/7).^15^ EPAS1-encoded HIF-2αis a critical driver of clear-cell renal cell carcinoma. Ablation of HIF-2αsignificantly reduces lipid peroxidation levels, demonstrating decreased cellular susceptibility to ferroptosis.^16^ In gliomas, the ferroptosis inducer 2-nitroimidazole specifically targets GSCs, enhancing radiation-induced DNA damage under hypoxic conditions.^17^ Elucidating hypoxia-induced ferroptosis mechanisms and their prognostic implications in gliomas will pave the way for novel therapeutic strategies.

## Materials and methods

### Sample and data

RNA sequences and clinical data were obtained from the CGGA (http://www.cgga.org.cn/.); the hypoxia gene set (M12975) was obtained from the GSEA(www.gsea-msigdb.org/.); and the ferroptosis marker genes was obtained from the FerrDb(www.zhounan.org/ferrdb/).

### Patterns of hypoxia by consensus clustering

Consensus clustering was used to identify distinct patterns of hypoxia. The number of clusters and their stability were determined using the ConsensuClusterPlus consensus clustering algorithm.^18^ Prognostic differences between clusters were measured (*p* < .05).

### Ferroptosis differences and prognostic genes

Based on different hypoxic mode clusters, we used the limma package to perform pairwise comparisons among the clusters and screen for genes that show statistically significant differences in all comparisons(|logFC| > 1, P-Value < .05). Prognostic analysis of differential genes was conducted(*p* < .05).

### Risk model and nomogram construction

The lasso method and Cox regression were used to construct risk models according to differential-prognostic genes. A receiver operating characteristics(ROC) curve was used to evaluate the efficacy of the risk model. Gene Set Enrichment Analysis(GSEA) was used to assess pathway enrichment. Univariate and multivariate Cox analyses were used to clinically assess the gliomas and establish a nomogram.

### Construction of the PPI and identification of key genes

The Protein-Protein Interaction(PPI) of HIF-1α and 23 prognostic genes was constructed on the String website. Key genes linked to HIF-1α with a confidence of 0.9 were selected.

### Immune correlation analysis

CIBERSORT was used to perform 22 immune cell infiltration analyses.^19^ Sample screening was performed with *p* < .05. Differences between the immune cells in each cluster and glioma prognosis were assessed(*p* < .05). The relationship between glioma prognosis, immune cell types, and key genes was analyzed.

## Results

### Samples and data

The CGGA database provided the training and validation datasets(Supplementary Table S1). In this study, 123 ferroptosis-related genes and 27 hypoxia-associated genes were analyzed.

### Consensus clustering

**Figure 1.**
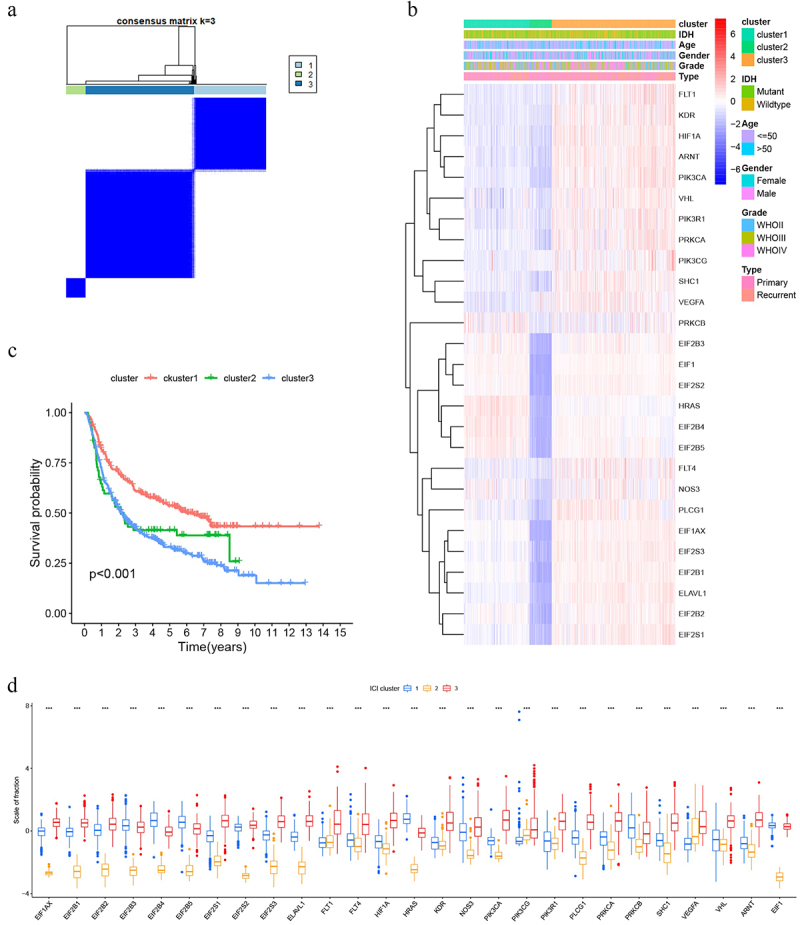
(a) Clustering based on hypoxia genes; (b) Hypoxia gene expression in response to different clinical conditions; (c) Prognostic differences between clusters; (d) Differences in hypoxia gene expression between the three clusters. All genes exhibited three expression gradients (high→low). Expression patterns aligned as follows^1^: EIF2B3, EIF2B4, EIF2B5, HRAs, PRKCB, EIF1: cluster 1→Cluster 3 →cluster 2^2^; EIF1AX, EIF2B1, EIF2B2, EIF2S1, EIF2S2, EIF2S3, ELAVL1, FLT4, HIF-1α, KDR, NOS3, PIK3CA, PLCG1, PRKCA, SHC1, VHL, ARNT: cluster 3→Cluster 1→Cluster 2^3^; FLT1, PIK3CG, VEGFA: cluster 3→Cluster 2→Cluster 1.

Samples segregated into three clusters exhibiting distinct prognostic outcomes (Figure 1a,b). We generated a clinical factor-responsive hypoxia gene expression heatmap (Figure 1c). Differential expression of all 27 hypoxia-associated genes was observed across these clusters (Figure 1d).

### Risk model

**Figure 2.**
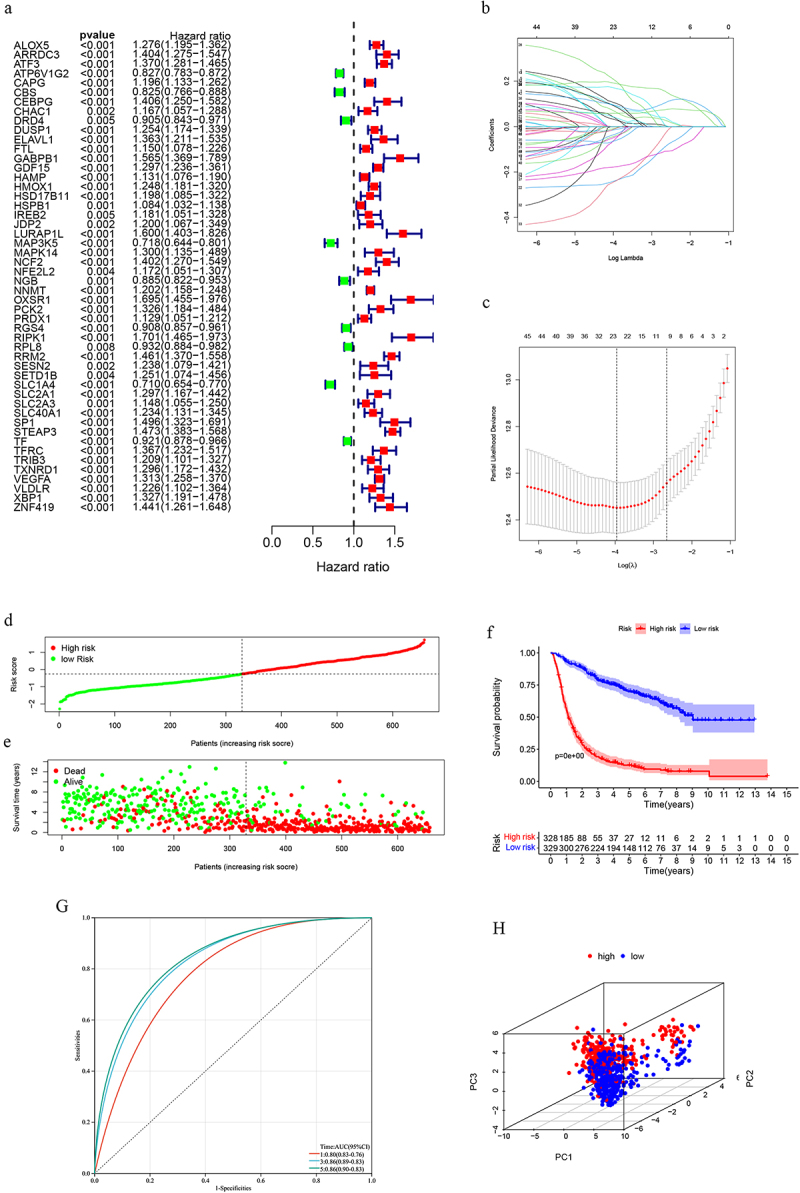
(a-c) Univariate prognostic analysis and Lasso regression; (d-e) Association between risk score and prognosis; (f) Survival curve based on the median value of the risk score; (g) The ROC curve assessing the predictive efficacy of the risk model; (h) Principal component analysis(PCA) to distinguish high- and low-risk groups.

Eighty-five ferroptosis-related differentially expressed genes (DEGs) were identified across the three clusters(Supplementary Table S2). From these, 23 prognosis-associated DEGs were selected to establish a risk model(Figure 2a-c). The risk score=

$$\overset{K}{\underset{i=1}{\sum }}\mathrm{CiEi}$$

where K, Ci, and Pi represented the number of signature genes, the coefficient index, and the gene expression level, respectively. The coefficient of each gene is shown in Table 1. The risk model demonstrated significant prognostic value for gliomas, as evidenced by risk distribution plots and survival curves (Figure 2d-f). Time-dependent receiver operating characteristic (ROC) analysis showed area under the curve(AUC) values of 0.80, 0.86, and 0.86 for 1-, 3-, and 5-year survival, respectively(Figure 2g). Principal component analysis (PCA) effectively distinguished between low- and high-risk groups(Figure 2h).

**Table 1.** The coefficient index of differential-prognostic genes.

Gene	Coef	Gene	Coef	Gene	Coef
ALOX5	0.030	NGB	0.089	SLC2A1	0.038
ATF3	0.066	OXSR1	0.203	SLC2A3	−0.010
CAPG	0.092	PRDX1	0.169	SLC40A1	−0.041
CEBPG	0.104	RGS4	−0.047	STEAP3	0.093
HAMP	−0.007	RPL8	−0.241	TF	0.040
HMOX1	−0.014	RRM2	0.190	VEGFA	0.058
MAP3K5	−0.239	SESN2	−0.062	XBP1	−0.034
NCF2	−0.057	SLC1A4	0.176		

### Risk model characteristics

**Figure 3.**
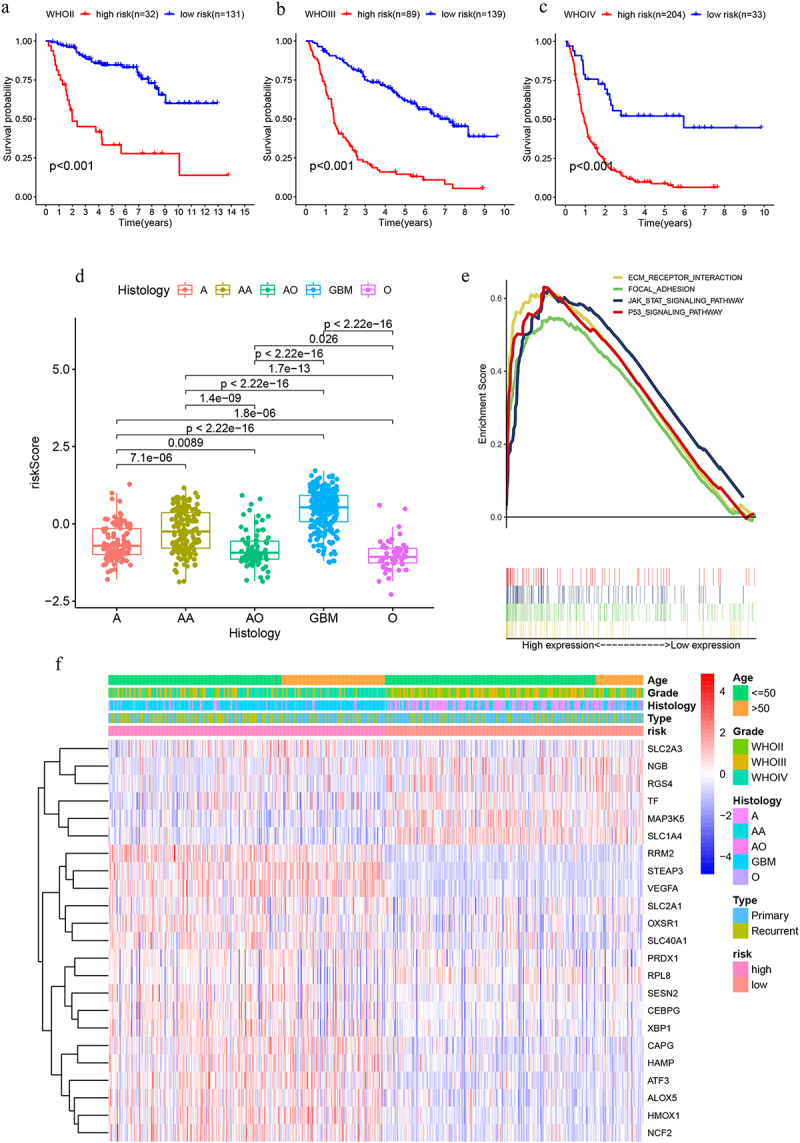
(a-c) The risk model in different grades of glioma; (d) Expression of risk scores in different glioma pathological types; (e) ECM receptor interaction, FOCAL adhesion, JAK-STAT, and P53 signaling pathway enhancement; (f) Expression of 23 genes in response to different clinical conditions.

The risk model demonstrated applicability across grade II, III, and IV gliomas (Figure 3a-c). Risk scores were significantly higher in glioblastoma (GBM) compared to oligodendroglioma(ODG)(Figure 3d). Significant enrichment was observed in ECM-receptor interaction, focal adhesion, JAK-STAT signaling, and p53 signaling pathways (Figure 3e). Figure 3f demonstrates differential expression patterns of the 23 model genes across clinical variables.

### Clinical risk assessment and nomogram construction

**Figure 4.**
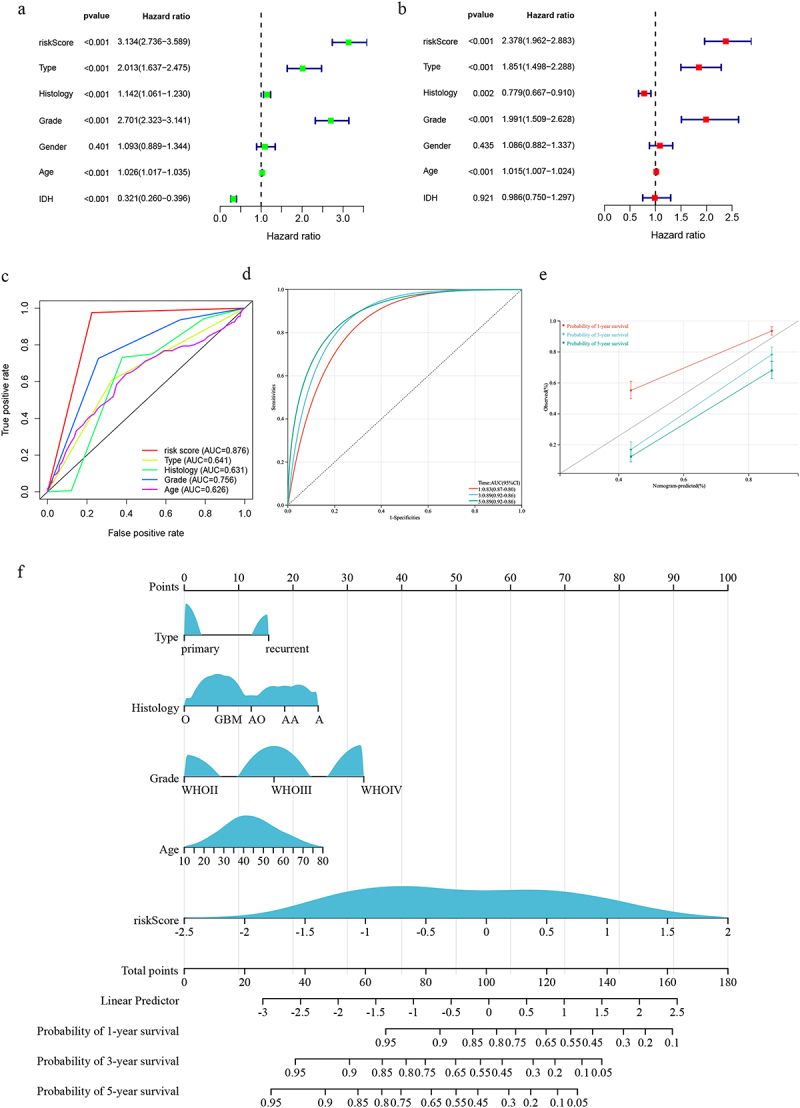
(a-b) Clinical risk assessment based on univariate and multivariate Cox analysis; (c) Comparison of the risk model to other clinical risk factors; (d) The AUC of 1, 3, and 5-year survival in the nomogram; (e) Calibration chart of 1, 3, and 5 year survival; (f) Nomogram.

Multivariate Cox regression identified the risk score, type, histology, grade, and age as independent prognostic factors for glioma patients (Figure 4a-b). The predictive accuracy of the risk model significantly outperformed other clinical parameters(*p* < .05; Figure 4c). A clinically applicable nomogram demonstrated robust prognostic stratification(C-index = 0.80; Figure 4d-f).

### PPI and identification of key genes

**Figure 5.**
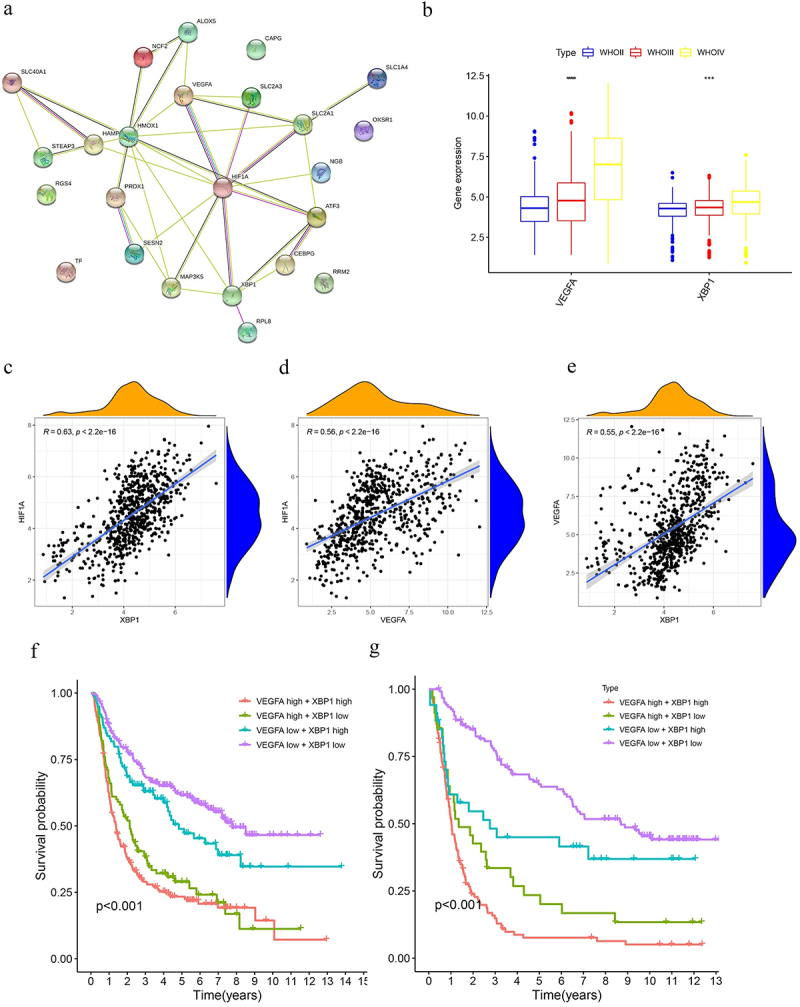
(a) PPI based on 23 prognostic ferroptosis and HIF-1α; (b) Expression of VEGFA and XBP1 in each grade of glioma; (c-e) correlation between VEGFA, XBP1, and HIF-1α; (f-g) Impact of XBP1 and VEGFA co-expression on glioma prognosis in the training and validation groups.

A protein-protein interaction (PPI) network was constructed using the 23 prognostic genes and HIF-1α (Figure 5a). Only VEGFA and XBP1 expression showed significant positive correlations with HIF1α expression at the highest confidence level. Both VEGFA and XBP1 expression increased significantly with glioma pathological grade (Figure 5b). Strong positive correlations were observed among VEGFA, XBP1, and HIF1α expression levels (Figure 5c-e). Notably, combined high expression of XBP1 and VEGFA was significantly associated with poor glioma prognosis (Figure 5f-g).

### Immune infiltration

**Figure 6.**
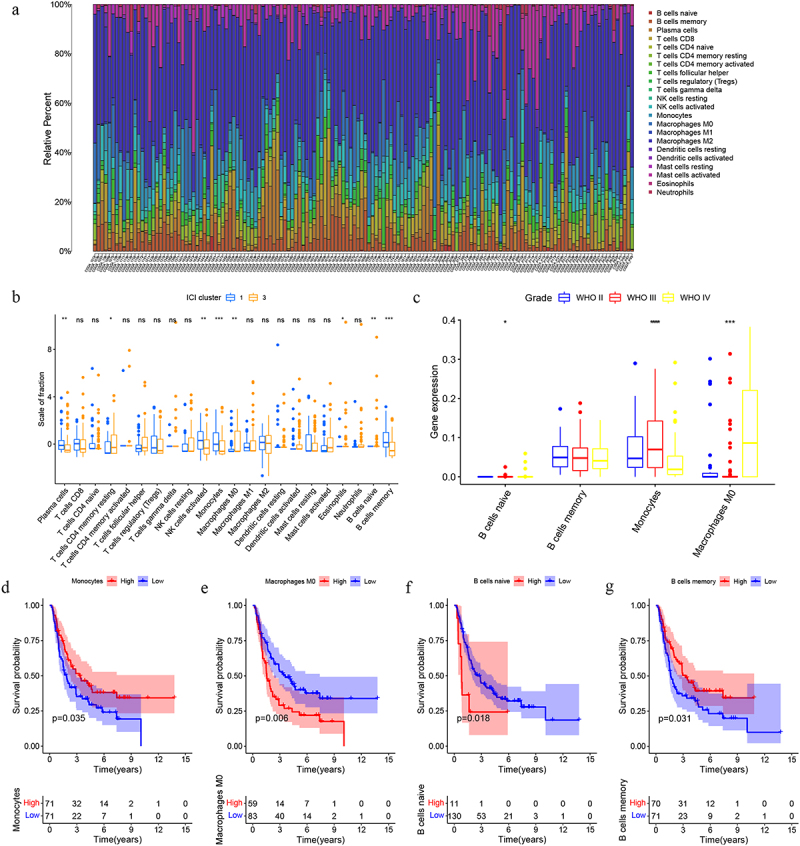
(a) Immune cell visualization of each sample; (b) The differential immune cell types in clusters 1 and 3; (d-g) Correlation between naïve B cells, memory B cells, monocytes, and M0 macrophages and glioma prognosis; (c) expression of four immune genes in different grades of glioma.

Figure 6a illustrated immune cell infiltration patterns across all samples. None of the 149 selected samples belonged to cluster 2. Significant differences in immune cell composition were observed between clusters 1 and 3 (Figure 6b). Prognostically relevant cell populations included naïve B cells, memory B cells, monocytes, and M0 macrophages (Figure 6d-g). Figure 6c displays glioma grade-dependent expression of four immune-related genes.

### Immune cell and key genes

**Figure 7.**
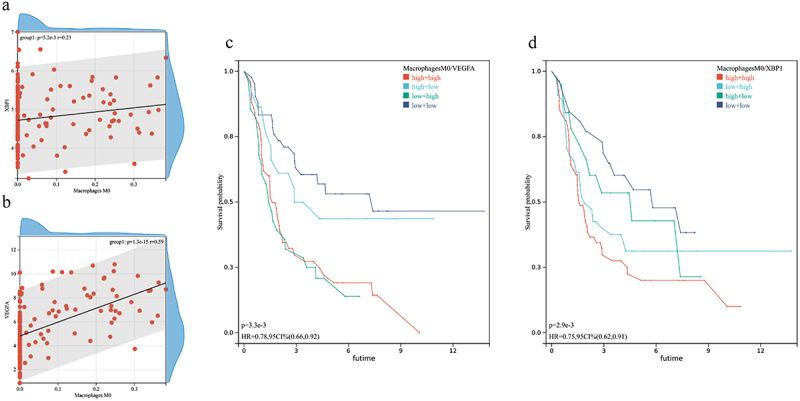
Correlation between M0 macrophages and VEGFA and XBP1 gene expression.

Only M0 macrophages demonstrated significant correlations with VEGFA and XBP1 expression (Figure 7a-b), and showed prognostic relevance for both genes (Figure 7c-d).

## Discussion

Hypoxia plays an important role in drug resistance and metastasis of malignant tumors, and can thus seriously impact prognosis. Targeting hypoxia-related key genes and signal pathways is a promising method for tumor treatment.^20^ Hypoxia-induced angiogenesis induces tumor growth, and significant progress has been made in the design of targeted drugs, such as Bevacizumab(BEV), that prevent angiogenesis.^21,22^ While BEV does not significantly improve the prognosis of GBM^23–25^，this drug is still considered an important treatment for GBM and combination therapy is viewed as a primary research direction. It is critical to find new drug targets in the event that multiple combination therapies fail.^26,27^

This study analyzed the impact of hypoxia on ferroptosis genes obtained from online databases. Hypoxia was found to significantly impact ferroptosis gene expression, and changes in ferroptosis correlated with glioma grade and prognosis. The PPI showed that VEGFA and XBP1 are key genes linked to HIF-1α and that co-expression affected glioma prognosis. These data indicate that XBP1 may be a new therapeutic target for use in combination with VEGFA. Immune infiltration analysis found that M0 macrophages may play an important role in potential combination treatment.

XBP1 is downstream of inositol-requiring enzyme 1(IRE1α) and is the key transcription factor in the unfolded protein response(UPR) that is important for cell survival in response to stress stimuli.^28^ UPR primarily functions to promote the correct folding of proteins, accelerate the degradation of misfolded proteins, and restore the stability of the endoplasmic reticulum.^29^ This function uses three signal pathways, inositol-requiring enzyme 1α and X-box binding protein 1(XBP1), protein kinase R(PKR)-like endoplasmic reticulum kinase(PERK), and actuating transcription factor 6(ATF6).^30^ The IRE1α-XBP1 branch is the most highly conserved endoplasmic reticulum stress(ERS) pathway. XBP1 can promote tumor immune evasion, inhibit autophagy and apoptosis, and accelerate tumor invasion and metastasis.^31–34^ An increase in expression of the hypoxia gene, HIF1-α, is the cellular response to hypoxic stress. HIF-1α combines with different hypoxia response genes to provide a material basis for the continuous growth, proliferation, and metastasis of tumor cells. After XBP1 is silenced under hypoxic conditions, tumor growth and survival decline, suggesting that XBP1 is a key component of cell survival.^35^ XBP1 is more highly expressed in glioma tumors than that in normal tissues and knocking out XBP1 can increase cell death under stress conditions.^36^ Studies show that XBP1 silencing reduces the viability and tumorigenicity of glioma cells by inhibiting expression of exocrine kinase II (HK2).^37^ In colon cancer, β-catenin inhibits XBP1s-mediated enhancement of HIF-1α target gene expression. In addition, β-catenin overexpression attenuates XBP1s-mediated cell survival under hypoxic conditions.^38^ In the current study, only XBP1 and VEGFA correlated with HIF-1α in PPI with the highest confidence, and co-expression was shown to significantly affect glioma prognosis. The internal regulation between XBP1 and VEGFA remains unclear. Interestingly, studies have found that depletion of XBP1 can block blood vessel formation^39^ and both XBP1 and VEGFA affect tumor progression by regulating immune cells.^40,41^ The results presented here show that M0 macrophages correlate with XBP1 and VEGFA expression and affect prognosis of the two genes. While the mechanism by which XBP1, VEGFA and immune cells function remains unclear, XBP1 may present a new target for use in combination with VEGFA to treat glioma.

## Conclusion

The findings of this study suggest that hypoxia can significantly change ferroptosis, and, in turn, affect glioma prognosis. XBP1 may be the new target for use with VEGFA in combination therapy.

## Supplementary Material

Supply materials table 1 clean.docx

Table S3.docx

Supply materials2.docx
