# Supplementary material for: Robust prediction of glioma prognosis by hypoxia-induced ferroptosis genes: VEGFA-XBP1 co-expression for salvage therapy
Source: Cancer Biol Ther. 2025 Jul 7;26(1):2529643. doi: 10.1080/15384047.2025.2529643 (PMC12239816; doi:10.1080/15384047.2025.2529643)
Supplement: Supply materials table 1 clean.docx [file KCBT_A_2529643_SM1934.docx]

| Table. 1Clinical characteristics | Training group  (n = 693) | Verification group  (n = 325) | *P* |
| --- | --- | --- | --- |
| Age | | | |
| <=50 | 505 | 237 | 1 |
| > 50 | 187 | 88 |  |
| Gender | | | |
| Female | 295 | 122 | 0.128 |
| Male | 398 | 203 |  |
| Type | | | |
| Primary | 422 | 229 | < 0.05 |
| Recurrence | 271 | 62 |  |
| Grade | | | |
| II | 188 | 103 | < 0.05 |
| III | 255 | 79 |  |
| IV | 249 | 139 |  |
| Histology | | | |
| A | 119 | 56 |  |
| AA | 152 | 62 |  |
| O | 60 | 52 | < 0.05 |
| AO | 82 | 12 |  |
| GBM | 249 | 139 |  |
| *IDH* mutation | | | |
| Yes | 356 | 174 | 0.752 |
| No | 286 | 146 |  |
| 1p19q codeletion | | | |
| Yes | 145 | 67 | 0.156 |
| No | 478 | 173 |  |
| MGMTp | | | |
| Yes | 315 | 155 | 0.057 |
| No | 227 | 147 |  |
| Radiation therapy | | | |
| Yes | 510 | 242 | 0.964 |
| No | 136 | 65 |  |
| TMZ therapy | | | |
| Yes | 486 | 191 | < 0.05 |
| No | 161 | 110 |  |
