# Supplementary material for: Robust prediction of glioma prognosis by hypoxia-induced ferroptosis genes: VEGFA-XBP1 co-expression for salvage therapy
Source: Cancer Biol Ther. 2025 Jul 7;26(1):2529643. doi: 10.1080/15384047.2025.2529643 (PMC12239816; doi:10.1080/15384047.2025.2529643)
Supplement: Table S3.docx [file KCBT_A_2529643_SM1933.docx]

| Table. 3 Differential genes in different clusteres | Differential genes |  |
| --- | --- | --- |
| High express→Low express |  |  |
| Cluster1→Cluster3→Cluster2 | EIF2B3,EIF2B4,EIF2B5,HRAS,PRKCB,EIF1 |  |
| Cluster3→Cluster1→Cluster2 | EIF1AX,FIF2B1,EIF2B2,EIF2S1,EIF2S2,EIF2S3,ELAVL1,FLT4,HIF1-A,KDR,NOS3,PIK3CA,PLCG1,PRKCA,SHC1,VHL,ARNT |  |
| Cluster3→Cluster2→Cluster1 | FLT1,PIK3CG,VEGFA, |  |
| Description: the expression of differential genes were divided into three categories according to the expression changes (cluster1-cluster3-cluster2;cluster3-cluster1-cluster2;cluster3-cluster2-cluster1), most of which are mainly concentrated in the cluster3-cluster1-cluster2 group. | |  |
