# Supplementary material for: Robust prediction of glioma prognosis by hypoxia-induced ferroptosis genes: VEGFA-XBP1 co-expression for salvage therapy
Source: Cancer Biol Ther. 2025 Jul 7;26(1):2529643. doi: 10.1080/15384047.2025.2529643 (PMC12239816; doi:10.1080/15384047.2025.2529643)
Supplement: Supply materials2.docx [file KCBT_A_2529643_SM1932.docx]

| Table. 2 Differential genes among clusteres | Differential gene(85) |
| --- | --- |
| Cluster2-Cluster1(64) | YWHAE GPX4 FTH1 ATF4 RPL8 ASNS UBC NFE2L2 HSPB1 STMN1 FTL PRDX1 CEBPG EIF2AK4 HSD17B11 DDIT3 SLC2A8 PSAT1 ELAVL1 HMGB1 RELA HERPUD1 MT3 GPT2 SLC3A2 DRD4 BNIP3 ACSF2 XBP1 KLHL24 RIPK1 MAPK14 EIF2S1 TSC22D3 SLC7A5 SESN2 SETD1B PCK2 TUBE1 TF CAPG ATP6V1G2 SRXN1 SLC1A4 ZNF419 CBS HAMP SLC2A6 JDP2 HBA1 TRIB3 IL33 CXCL2 ALB CHAC1 ALOX5 NGB HMOX1 DDIT4 ATF3 NNMT VEGFA SLC2A3 RGS4 |
| Cluster3-Cluster1(32) | GABPB1 SP1 OXSR1 TXNRD1 IREB2 ELAVL1 ARRDC3 HMGB1 EIF2S1 GPX4 TUBE1 VEGFA SLC40A1 MAFG TFRC BNIP3 DRD4 MT3 RPL8 KLHL24 TXNIP SLC2A1 RRM2 ACSF2 IL33 NGB HSPB1 DUSP1 CAPG TF STEAP3 HBA1 |
| Cluster3-Cluster2(69) | YWHAE FTH1 ATF4 GPX4 NFE2L2 ELAVL1 ASNS HMGB1 RPL8 CEBPG HSD17B11 MAPK14 STMN1 RELA UBC BNIP3 PSAT1 EIF2S1 FTL SETD1B TUBE1 XBP1 EIF2AK4 GPT2 PRDX1 DDIT3 HERPUD1 HSPB1 RIPK1 SLC2A8 SLC7A5 SRXN1 IREB2 SLC3A2 ARRDC3 TXNRD1 AGPAT3 SESN2 ZNF419 IL33 MAFG SLC40A1 PCK2 TSC22D3 GABPB1 ATP6V1G2 MT3 JDP2 TXNIP VLDLR LURAP1L SLC7A11 CBS SLC1A4 TRIB3 TFRC SLC2A1 ALOX5 ATF3 HMOX1 ACSF2 DRD4 HAMP MAP3K5 CXCL2 TF CAPG NCF2 GDF15 |
